# The Role of Dynamic Contrast-Enhanced MRI in a Child with Sport-Induced Avascular Necrosis of the Scaphoid: A Case Report and Literature Review

**DOI:** 10.1155/2016/7898090

**Published:** 2016-07-26

**Authors:** Baris Beytullah Koc, Martijn Schotanus, Bob Jong, Pieter Tilman

**Affiliations:** ^1^Department of Orthopedic Surgery, Zuyderland Medical Centre, Dr. H. van der Hoffplein 1, 6162 BG Sittard-Geleen, Netherlands; ^2^Department of Radiology, Zuyderland Medical Centre, Dr. H. van der Hoffplein 1, 6162 BG Sittard-Geleen, Netherlands

## Abstract

Avascular necrosis (AVN) of the scaphoid in children is very rare and there is currently no consensus when conservative or operative treatment is indicated. A 10-year-old boy, practicing karate, presented with acute pain in his left wrist after falling on the outstretched hand. Imaging showed a scaphoid waist fracture with signs of an ongoing AVN. The diagnosis of AVN was confirmed with signal loss of the scaphoid on MRI T1. A dynamic contrast-enhanced MRI was performed for further assessment of the proximal pole vascularity and treatment planning. As dynamic contrast-enhanced MRI showed fair perfusion of the proximal pole, an adequate healing potential with conservative treatment was estimated. We achieved union and good function with cast immobilization for fourteen weeks. This case study showed dynamic contrast-enhanced MRI to be a valuable tool in assessing whether conservative or operative treatment is indicated to achieve union and good functional outcome.

## 1. Introduction

Cases of avascular necrosis (AVN) of the scaphoid are very rare with only six cases regarding the management of AVN in children reported [1–3]. Because of the limited reports there is currently no consensus when conservative or operative treatment is indicated. We report a case of a child with sport-induced AVN of the scaphoid in whom a dynamic contrast-enhanced MRI was performed for further treatment planning. The patient and his parents were informed about this report and agreed to its publication.

## 2. Case Presentation

**Figure 1:**
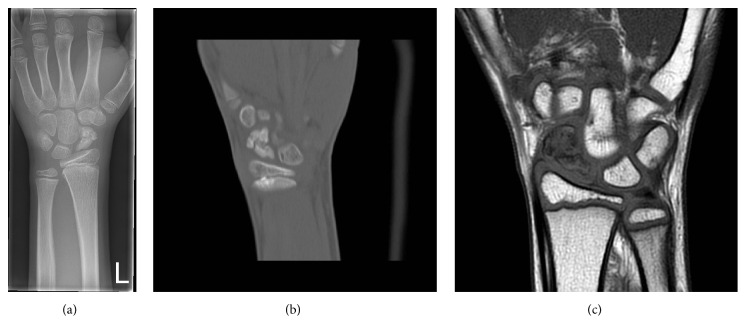
(a) Plain radiograph at acute presentation showing a fracture of the scaphoid waist with sclerosis, central cystic bone alteration, and deformity of the proximal pole. (b) CT scan 1 week after the initial injury showing a more pronounced central cystic bone alteration and irregularity of the fracture border. (c) MRI T1 showing signal loss of the whole scaphoid.

**Figure 2:**
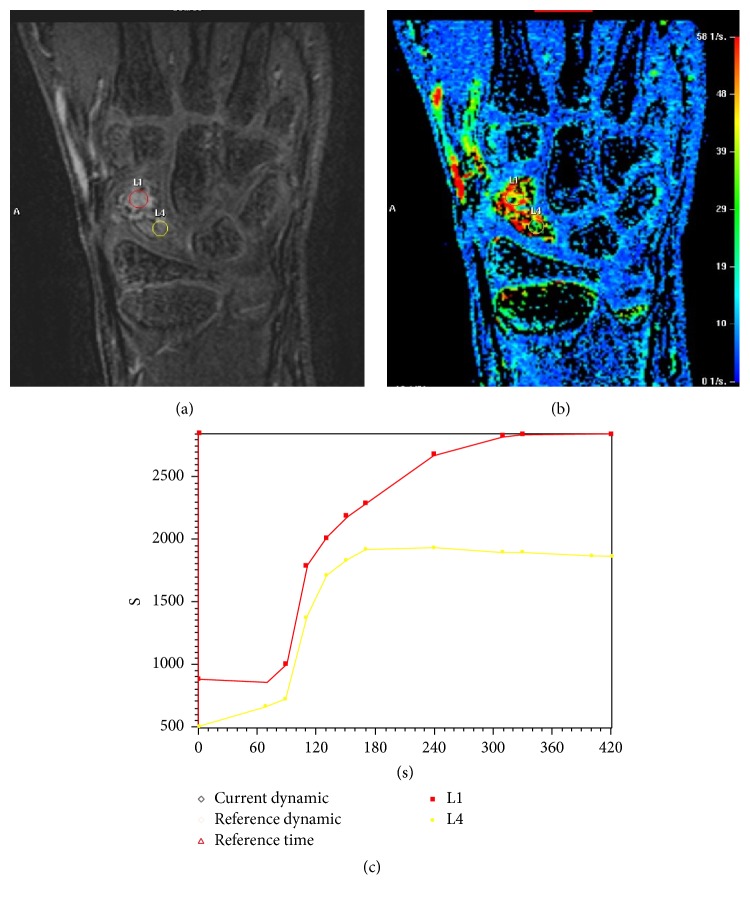
To assess the vascularity with dynamic contrast-enhanced MRI, a region of interest was placed on the proximal and distal scaphoid poles and time-signal intensity curves were recorded. The dynamic contrast-enhanced MRI showed fair perfusion of the proximal pole with account of measuring associated fibroblasts in the cystic alteration in the distal pole. The time-signal intensity curve on the proximal pole (yellow curve) was lower than the distal pole (red curve) with a maximum enhancement of 50%.

**Figure 3:**
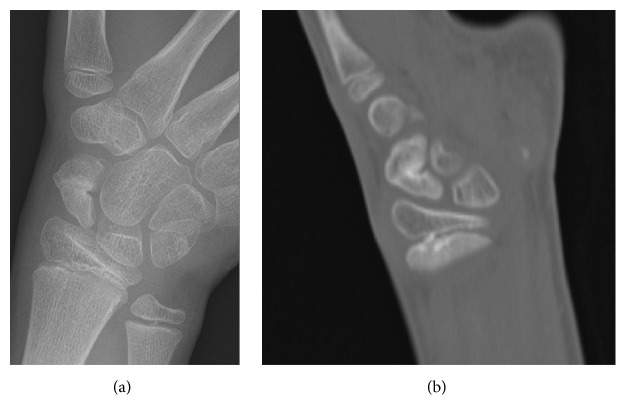
(a) Plain radiograph at the end of cast immobilization showing improved consolidation. (b) CT scan be performed one month after the end of cast immobilization showing union of the fracture.

A 10-year-old boy, practicing karate since the age of six, presented with acute pain in his left wrist after falling on the outstretched hand. On physical examination, there was tenderness in the anatomical snuffbox and a painful range of motion of the wrist. Plain radiograph showed a fracture of the scaphoid waist with sclerosis, central cystic bone alteration, and deformity of the proximal pole suggesting an ongoing AVN (Figure 1(a)). Additional CT scan supported the radiographic findings with a more pronounced central cystic bone alteration and irregularity of the fracture border (Figure 1(b)). The diagnosis of AVN was confirmed with signal loss of the scaphoid on MRI T1 (Figure 1(c)). A dynamic contrast-enhanced MRI was performed for further assessment of the proximal pole vascularity and treatment planning. Therefore, a region of interest was placed on the proximal and distal scaphoid poles. Time-signal intensity curves were recorded and were considered to represent the degree of vascularity. The time-signal intensity curves are classified into good, fair, or poor vascularity based on their shape and maximal enhancement comparing proximal pole with the distal pole [4, 5]. In this study, the time-signal intensity curve on the proximal pole was lower than the distal pole with a maximum enhancement of 50%, defining fair perfusion of the proximal pole. The fair perfusion of the proximal pole on dynamic contrast-enhanced MRI was with the account of measuring associated fibroblasts in the cystic alteration in the distal pole (Figure 2). Because of fair perfusion of the proximal pole, an adequate healing potential with conservative treatment was estimated. The wrist was immobilized with a short arm cast for fourteen weeks and at the end of cast immobilization the patient was pain-free and had no tenderness and there was no restriction in range of motion. Plain radiograph showed improved consolidation and CT scan performed one month later confirmed union of the fracture (Figures 3(a) and 3(b)).

## 3. Discussion

Scaphoid fractures are rare, accounting for 0.4% of all fractures in the pediatric population [6]. Contrary to adults, scaphoid fractures in children involved mainly the distal pole [6]. However, it is believed that the grown intensive sport participation among children has caused a shift in the presentation of pediatric scaphoid fractures, resembling the adult pattern with mainly involvement of the scaphoid waist [7]. The retrograde perfusion of the scaphoid makes the scaphoid waist more susceptible for AVN than the distal pole [8]. Thereby, the diagnosis of AVN of the scaphoid is made by signal loss on MRI T1 [9]. In this case study, AVN was confirmed in the presence of a scaphoid waist fracture. Although a recent traumatic event occurred, the radiographic findings indicate the result of a longer existing disorder. A sport-induced stress fracture of the scaphoid waist is believed to have contributed to AVN of the scaphoid in a child practicing an intensive sport as karate. Stress fracture of the scaphoid is the result of repetitive dorsiflexion of the wrist, with the waist as the weakest point in the scaphoid [10]. Furthermore, the risk of sport-induced AVN is supposed to increase as a result of the grown sport participation among children [7]. However, there is currently no consensus when conservative or operative treatment is indicated. Gunal and Altay reported good results of two children with AVN treated by immobilization for several weeks [2]. Waters and Stewart achieved union and good function with vascularized bone grafting and internal fixation after failure of immobilization [3]. Barthel et al. reported one case of AVN treated with vascularized bone grafting and internal fixation, obtaining union and pain relief [1]. The initial diagnosis and management in these studies were based on signal loss of the scaphoid on MRI T1 [2–4]. In our case study, a dynamic contrast-enhanced MRI was used for further treatment planning, as it is believed to be superior to contrast-enhanced MRI in assessing the vascularity of the scaphoid [4]. As dynamic contrast-enhanced MRI showed fair perfusion of the proximal pole, an adequate healing potential with conservative treatment was estimated. Subsequently, we achieved union and good functional outcome with cast immobilization for fourteen weeks.

In conclusion, this study showed dynamic contrast-enhanced MRI to be a valuable tool in assessing whether conservative or operative treatment is indicated to achieve union and good functional outcome in a child with sport-induced AVN of the scaphoid. If dynamic contrast-enhanced MRI had shown poor perfusion of the proximal pole, primary operative intervention would be indicated. Further studies are necessary to confirm the decision algorithm used in this study.
